# Kinetics and Cellular Site of Glycolipid Loading Control the Outcome of Natural Killer T Cell Activation

**DOI:** 10.1016/j.immuni.2009.03.022

**Published:** 2009-06-19

**Authors:** Jin S. Im, Pooja Arora, Gabriel Bricard, Alberto Molano, Manjunatha M. Venkataswamy, Ian Baine, Elliot S. Jerud, Michael F. Goldberg, Andres Baena, Karl O.A. Yu, Rachel M. Ndonye, Amy R. Howell, Weiming Yuan, Peter Cresswell, Young-tae Chang, Petr A. Illarionov, Gurdyal S. Besra, Steven A. Porcelli

**Affiliations:** 1 Department of Microbiology and Immunology , Albert Einstein College of Medicine, Bronx, NY 10461, USA; 2 Department of Medicine, Albert Einstein College of Medicine, Bronx, NY 10461, USA; 3 Department of Chemistry, University of Connecticut, Storrs, CT 06269-3060, USA; 4 Department of Immunobiology and Howard Hughes Medical Institute, Yale University School of Medicine, New Haven, CT 06520-8011, USA; 5 Department of Chemistry and NUS MedChem Program of the Life Sciences Institute, National University of Singapore, Singapore 117543, Republic of Singapore; 6 School of Biosciences, University of Birmingham, Edgbaston, Birmingham B15 2TT, UK

**Keywords:** MOLIMMUNO, CELLIMMUNO

## Abstract

CD1d-restricted natural killer T cells (NKT cells) possess a wide range of effector and regulatory activities that are related to their ability to secrete both T helper 1 (Th1) cell- and Th2 cell-type cytokines. We analyzed presentation of NKT cell activating α galactosylceramide (αGalCer) analogs that give predominantly Th2 cell-type cytokine responses to determine how ligand structure controls the outcome of NKT cell activation. Using a monoclonal antibody specific for αGalCer-CD1d complexes to visualize and quantitate glycolipid presentation, we found that Th2 cell-type cytokine-biasing ligands were characterized by rapid and direct loading of cell-surface CD1d proteins. Complexes formed by association of these Th2 cell-type cytokine-biasing αGalCer analogs with CD1d showed a distinctive exclusion from ganglioside-enriched, detergent-resistant plasma membrane microdomains of antigen-presenting cells. These findings help to explain how subtle alterations in glycolipid ligand structure can control the balance of proinflammatory and anti-inflammatory activities of NKT cells.

## Introduction

Natural killer T cells (NKT cells) comprise several unconventional T cell subsets that coexpress T cell antigen receptors (TCRs) and various receptors that are prominently expressed by NK cells (Godfrey et al., 2004). Among the several subsets of NKT cells, the most well studied are those that express an invariant TCRα chain, which are known as type 1 or invariant NKT cells (iNKT cells). These recognize glycolipid antigens presented by the nonpolymorphic MHC class I-like molecule CD1d (Bendelac et al., 2007). Upon activation, iNKT cells release IFN-γ and other proinflammatory cytokines, as well as activate a variety of other leukocytes to express effector functions. Through a combination of these and other activities, iNKT cells can exert major effects on early and delayed adaptive immunity to tumors and to a wide variety of infectious pathogens (Crowe et al., 2005; Behar and Porcelli, 2007). In addition, iNKT cells also contribute to maintenance of immune tolerance (Yu and Porcelli, 2005; Jiang et al., 2007). This is believed to be related at least in part to the innate programming of iNKT cells to produce a variety of anti-inflammatory or regulatory cytokines, including high amounts of several T helper 2 (Th2) cell-associated cytokines such as IL-4, IL-5, and IL-13 (Im et al., 2006; Sakuishi et al., 2007).

A major advance in iNKT cell research was the discovery of specific glycolipid ligands that are recognized by these cells when presented by CD1d. The prototypical iNKT cell activator is a synthetic form of α-galactosylceramide with a C18 phytosphingosine base and a fully saturated C26 N-linked fatty acyl chain, here referred to as αGalCer-C26:0 (Kawano et al., 1997). Many studies have shown that αGalCer-C26:0 is a potent activator of both mouse and human iNKT cells and that it elicits production of both Th1 cell- and Th2 cell-type cytokines. There has been substantial effort toward identifying activating ligands that can more precisely focus the outcome of the iNKT cell response, and several studies have shown that alterations of the basic lipid structure of αGalCer-C26:0 can lead to striking changes in the patterns of cytokine production (Miyamoto et al., 2001; Schmieg et al., 2003; Goff et al., 2004; Yu et al., 2005; Fujio et al., 2006). The ability of an iNKT cell-activating ligand to induce a bias toward production of cytokines associated with Th2 cell responses was first described for the αGalCer analog designated OCH, in which the sphingoid base was truncated to C9 and the fatty acid chain was slightly shortened to C24:0 (Miyamoto et al., 2001). A similar cytokine-biasing property was described by our studies of an αGalCer derivative containing an 11,14-cis-diunsaturated C20 fatty acid (αGalCer C20:2) (Yu et al., 2005) and by another study of αGalCer analogs with truncated fatty acyl chains (Goff et al., 2004). Conversely, a few derivatives of αGalCer have been described that polarize the cytokine response in the opposite direction, with IFN-γ predominating over IL-4 production (Schmieg et al., 2003; Fujio et al., 2006; Chang et al., 2007). These compounds are of interest as selective activators of iNKT cell functions, and such activators may lend themselves to a variety of therapeutic applications (Miyake and Yamamura, 2005). However, the mechanisms by which structural variants of αGalCer induce selective activation of iNKT cell functions are not clearly understood, and this imposes limits on rational development of optimized iNKT cell activators for specific applications.

In the current study, we used a panel of analogs of αGalCer that induced preferential production of Th2 cell-type cytokines to explore the mechanism by which these compounds induce these biased cytokine responses. Although these αGalCer analogs showed marked variability in their avidities for the TCRs of iNKT cells, they consistently displayed the ability to rapidly load onto CD1d molecules directly at the cell surface of antigen-presenting cells (APCs). This rapid loading of CD1d could be directly visualized with a recently developed monoclonal antibody specific for the complex formed by the binding of αGalCer to murine CD1d. Most notably, αGalCer-CD1d complexes containing Th2 cell-type cytokine-biasing analogs showed a marked depletion in their localization to ganglioside-enriched, detergent-resistant microdomains in the plasma membranes of APCs, providing a mechanistic link between the structure of CD1d-binding glycolipids and their ability to preferentially induce immunoregulatory activities of iNKT cells.

## Results

### Th2 Cell-Type Cytokine-Biasing αGalCer Analogs for Murine iNKT Cells

**Figure 1:**
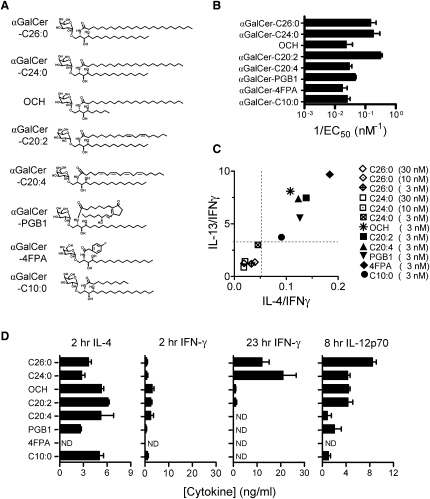
Synthetic Analogs of αGalCer (A) Structures and nomenclature of iNKT cell-activating αGalCer analogs used in this study. The nomenclature used in most cases is based on the identity of the amide-linked acyl group in the ceramide moiety. Acyl carbon chain length and number of unsaturations are indicated by the names of most analogs, with exceptions being OCH (which has a C24 saturated acyl chain) and the compounds with amide-linked prostaglandin B1 (PGB1) or 4-fluorophenyl acetate (4FPA) groups. The phytosphingosine base is 18 carbons in length for all analogs except OCH, which has a nine-carbon base. (B) Hybridoma DN3A4.1-2 (Vβ8.2^+^) was stimulated with each of the αGalCer analogs at a range of concentrations with JAWS II cells as APCs, and the amounts of IL-2 production were analyzed. Potencies of glycolipids are shown as 1/EC_50_ (as defined in Figure S1). The graph shows mean values ± SD, and the results shown are representative of three independent experiments. (C) Ratios of IL-4 to IFN-γ (x axis) plotted against the ratios of IL-13 to IFN-γ (y axis) for culture supernatants of BALB/c splenocytes stimulated with glycolipids for 48 hr. Ratios for the C26:0 and C24:0 analogs are shown for stimulation with a range of glycolipid concentrations (3, 10, and 30 nM). Other analogs are shown at one concentration (3 nM) for clarity, although similar trends were observed at higher glycolipid concentrations (Figure S2). Data are representative of three experiments. (D) C57BL/6 mice (four mice per group) received a single injection i.p. of four nanomoles of each glycolipid, and blood samples were obtained at 2, 8, and 23 hr. Concentrations of IFN-γ, IL-4, and IL-12p70 were determined in serum by capture ELISA. “ND” stands for not detected. The graphs show mean ± SD for triplicate values, and results shown were representative of two independent experiments.

Eight synthetic αGalCer analogs (Figure 1A) were selected for use in this study from two synthetic libraries of N-acyl variants of the basic α-galactosyl C18 phytosphingosine structure (Yu et al., 2005; Li et al., 2007). Among these were two compounds that induce mixed Th1 and Th2 cell-type cytokine responses, αGalCer-26:0 and the closely related αGalCer-C24:0 variant (Forestier et al., 2007). The six analogs that induced predominantly Th2 cell-type cytokine responses included the previously described OCH and αGalCer-C20:2 and -C20:4 compounds (Miyamoto et al., 2001; Yu et al., 2005) and three additional N-acyl derivatives of the α-galactosyl C18 phytosphingosine base. These compounds were selected because of their ability to potently activate mouse iNKT cell hybridomas (Figure 1B and Figure S1 available online), as well as their induction of increased ratios of IL-13 or IL-4 to IFN-γ from mouse splenocytes (Figure 1C and Figure S2). In vivo analysis revealed that injection of mice with five of the six Th2 cell-type cytokine-biasing analogs produced concentrations of circulating IL-4 that were similar to or greater than those produced by injection of αGalCer-C26:0 and -C24:0 compounds (Figure 1D), but in all cases these compounds elicited low or undetectable serum IFN-γ either early (2 hr) or late (23 hr) after injection. As we previously reported (Forestier et al., 2007), circulating IL-12p70 concentrations in vivo did not correlate with the Th2 cell-type cytokine-biasing properties of either OCH or αGalCer-C20:2, although other analogs with a similar bias toward Th2 cell-type cytokine production showed a tendency toward reduced IL-12p70 production (Figure 1D). One of the analogs studied, αGalCer-4FPA, showed minimal activity in vivo in C57BL/6 mice (Figure 1D) but was included because of its pronounced Th2 cell-type biasing effect in vitro. Most of the αGalCer analogs in this panel were also able to strongly stimulate human iNKT cells in vitro (Figure S3).

### Murine and Human iNKT Cell TCR Interactions with αGalCer Analogs

**Figure 2:**
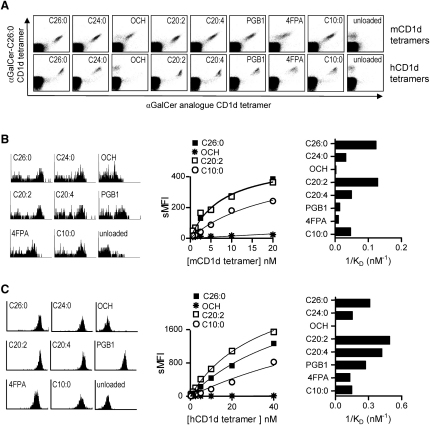
Characterization of iNKT Cell TCR Interactions with αGalCer Analogs (A) Flow cytometry analysis of human and murine iNKT cells with αGalCer analog-CD1d tetramers. In the top row, murine iNKT cells from C57BL/6 splenocytes were costained with αGalCer analog-loaded or unloaded mCD1d tetramers conjugated with PE (x axis) and αGalCer-C26:0-loaded-mCD1d tetramers conjugated with APC (y axis). In the bottom row, human PBMCs were similarly costained with human glycolipid-loaded CD1d tetramers. (B) Binding avidity of glycolipid-mCD1d complexes to murine iNKT cell TCRs. Histograms at left show tetramer staining of NK1.1^+^ CD3^+^ T cells in C57BL/6 splenocytes with glycolipid-loaded tetramers at 20 nM. Mean fluorescence intensity (MFI) for staining of this population with tetramers at various concentrations is plotted (center), and the equilibrium dissociation constants (K_D_) were determined as the concentrations of glycolipid-mCD1d complexes required to yield 50% of maximal binding. Values are plotted as 1/K_D_, which is directly proportional to TCR avidity (right). (C) Similar experiments as in (B) for binding of αGalCer-hCD1d tetramers to a human iNKT cell clone (CD4^+^ clone HDD11). All results shown were representative of two independent experiments.

The interactions of iNKT cell TCRs with CD1d molecules loaded with αGalCer analogs were analyzed with fluorescent murine and human CD1d tetramers. This demonstrated that iNKT cells recognizing each analog were also strongly reactive with αGalCer-C26:0, although staining patterns in some cases indicated differences in relative TCR avidities (Figure 2A). Equilibrium binding studies for measuring TCR avidities were carried out by incubation of human or murine iNKT cells with varying concentrations of αGalCer-analog-loaded tetramers and determining relative levels of cell-associated fluorescence (Stanic et al., 2003; Yu et al., 2005). A wide range of TCR avidities was observed for mouse CD1d (mCD1d) and human CD1d (hCD1d) complexes formed with different Th2 cell-type cytokine-biasing αGalCer analogs (Figures 2B and 2C). Notably, complexes loaded with some of these analogs showed avidities that were equal to or exceeded those of αGalCer-C26:0 or -C24:0 loaded complexes, arguing that low TCR avidity was not the main or sole determinant responsible for altered cytokine responses.

### APC Requirement and Kinetic Features of Presentation of αGalCer Analogs

**Figure 3:**
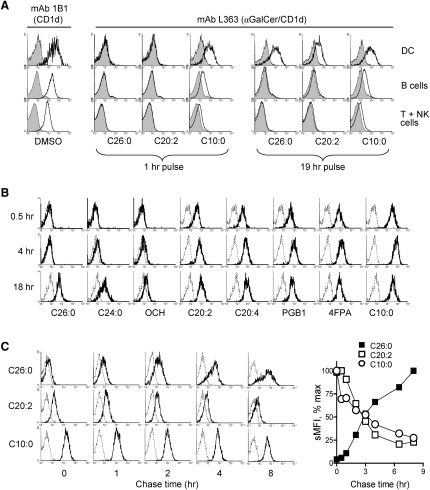
Direct Analysis of Presentation of αGalCer Analogs with Complex-Specific mAb Staining (A) Mouse splenocytes cultured with either vehicle only (0.02% DMSO) or with the indicated αGalCer analogs (100 nM) were harvested after 1 hr or 19 hr of incubation and stained with mAb L363 or anti-CD1d mAb 1B1 and with mAbs specific for CD11c, CD19, and F4/80 (Figure S4). Filled histograms show L363 staining of cells cultured with vehicle only, and open histograms show cells cultured with indicated glycolipids. At the left, the total CD1d staining with mAb 1B1 (open histograms) compared to isotype control mAb (filled histograms) are shown on cells cultured for 19 hr with DMSO only. (B) JAWS II cells were incubated with glycolipids (250 nM) for various times ranging from 0.5–18 hr as indicated, stained with mAb L363, and analyzed by flow cytometry. Dashed line histograms show L363 staining of cells incubated with medium only, and bold histograms show L363 staining of cells incubated with αGalCer analogs. (C) JAWS II cells were pulsed with indicated αGalCer analogs (500 nM) for 1.5 hr, washed extensively, and recultured at 37°C for various chase times. Upon harvesting, cells were placed on ice and subsequently stained with mAb L363 for flow cytometry analysis. Histogram overlays show L363 staining of cells pulsed initially with vehicle only (DMSO, dashed histograms) or with αGalCer analog (bold histograms). The graph on the right shows MFI of L363 staining at each chase time represented as percent of the maximum value measured during the course of the experiment. All results shown (A, B, and C) were representative of at least two independent experiments.

A previous study suggested that an iNKT cell response biased toward production of Th2 cell-type cytokines could result from preferential presentation of αGalCer by B lymphocytes (Bezbradica et al., 2005). We studied this possibility directly by using a monoclonal antibody (L363) that specifically recognizes complexes of αGalCer bound to mCD1d (Yu et al., 2007). Mouse splenocytes were cultured with either αGalCer-C26:0 or representative Th2 cell-type cytokine-biasing analogs, and L363 staining was assessed on various cell types (Figure 3A). This revealed that all αGalCer analogs were presented most prominently on CD11c^+^ DCs, and only one of the two analogs that induced a Th2 cell-type cytokine response showed clearly detectable presentation by B cells even after prolonged incubation (19 hr). However, the extremely early appearance of L363 staining on cells exposed to αGalCer-C10:0 suggested that rapid kinetics of presentation might be a key feature of αGalCer analogs that induce a bias toward Th2 cell-type cytokines. This was investigated further with more detailed kinetic studies in which immortalized mouse DC line JAWS II was cultured with each glycolipid and assessed for L363 surface staining at 0.5, 4, and 18 hr (Figure 3B). There was a striking correlation between rapid kinetics of glycolipid association with mCD1d and the ability to induce a Th2 cell-type cytokine response. For analogs that induced a mixed cytokine response (αGalCer-C26:0 and -C24:0), strong L363 staining was detected only at 18 hr. In contrast, most analogs inducing Th2 cell-type cytokine responses gave strong L363 staining by 0.5 hr, which increased modestly or not at all with longer incubation. The single exception was OCH, which in general gave very weak L363 reactivity, although this was also apparent by 4 hr. A pulse-chase analysis revealed that mCD1d-αGalCer surface complexes formed slowly with αGalCer-C26:0 and continued to accumulate throughout the 8 hr chase (Figure 3C), whereas with αGalCer-C20:2 or -C10:0, the complexes formed rapidly and began declining immediately during the chase period. Experiments using responses of iNKT cells to analyze the kinetics of cell-surface appearance gave analogous results (Figure S5). Thus, although our findings did not provide evidence for presentation by different APC types as a major factor accounting for the altered cytokine responses to different analogs of αGalCer, they revealed a consistent tendency for glycolipids that induced Th2 cell-type biased cytokine responses to be presented with more rapid kinetics compared to αGalCer analogs that induced mixed Th1 and Th2 cell-type cytokine responses.

### Direct Imaging of CD1d Loading with αGalCer Analogs

**Figure 4:**
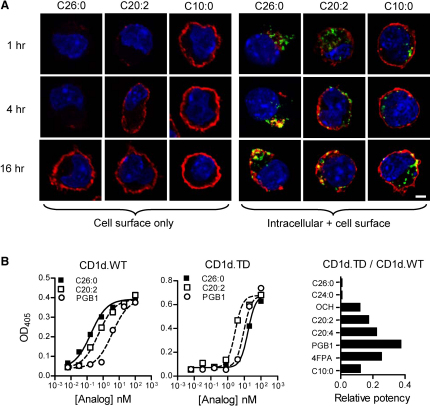
Cell Surface versus Intracellular Loading of mCD1d with Different αGalCer Analogs (A) On the left, surface accumulation of CD1d-αGalCer complexes was studied with JAWS II cells harvested after incubation with the indicated glycolipids for 1, 4, or 16 hr and then stained without permeabilization with mAb L363 (red). Cells were then fixed, stained with DAPI for nuclei visualization (blue), and analyzed by confocal microscopy. On the right, the intracellular formation and trafficking of CD1d-αGalCer complexes were analyzed by treatment of JAWS II cells with αGalCer analogs for 1, 4, and 16 hr followed by fixation and permeabilization. The cells were stained for CD1d-αGalCer complexes (L363, red), late endosomes and lysosomes (anti-LAMP-1, green), and nuclei (DAPI, blue). Results are representative of three separate experiments. The scale bar represents 10 μm. (B) Murine iNKT cell hybridoma cells (DN3A4.1-2) were stimulated with αGalCer analogs. As APCs for these stimulations, we used A20 cells transfected with wild-type CD1d (CD1d.WT) or cytoplasmic-tail-deleted CD1d (CD1d.TD). Supernatant concentrations of IL-2 release were determined after 12 hr. IL-2 production over a range of concentrations is shown for αGalCer-C26:0 and two analogs that produce a Th2 cell-type cytokine bias (αGalCer-C20:2 and -PGB1). The bar graph on the right shows relative potencies [(EC_50_ with presentation by CD1d.WT)/(EC_50_ with presentation by CD1d.TD)] of each glycolipid presented by CD1d.TD versus CD1d.WT.

To visualize the kinetics and sites of mCD1d loading by αGalCer analogs in live cells, we analyzed intact JAWS II cells for surface staining with mAb L363 after 1, 4, or 16 hr of incubation with αGalCer-C26:0 or with either of two Th2 cell-type cytokine-biasing analogs (αGalCer-C20:2 or -C10:0) (Figure 4A). Strong staining of the plasma membrane was seen after 16 hr of incubation in all cases. However, only αGalCer-C20:2 or -C10:0 showed L363 staining at earlier time points, consistent with the rapid kinetics of loading observed by flow cytometry. To image earlier steps in glycolipid loading, we permeabilized and fixed cells prior to staining with L363 and with an antibody to the late endosomal and lysosomal marker LAMP-1 (Figure 4A). After incubation for 1 hr with glycolipids, L363 staining was observed at the plasma membrane for αGalCer-C20:2 and -C10:0, and no colocalization with LAMP-1 was present. Colocalization with LAMP-1 was observed for mCD1d complexes formed with these analogs only at the latest time point studied (16 hr), suggesting that they arrived at late endocytic compartments via recycling from the plasma membrane. In contrast, L363 staining of cells pulsed for 1 hr with αGalCer-26:0 was observed only in a punctate intracellular distribution that was completely distinct from LAMP-1 staining, indicating that the initial association with mCD1d occurred in an endocytic compartment separate from typical late endosomes and lysosomes. At 4 hr, these complexes began to colocalize with LAMP-1, and only at 16 hr could clear surface staining be seen along with the persistent intracellular staining in LAMP-1^+^ compartments (Figure 4A).

The lack of detectable intracellular mCD1d loading prior to cell-surface mCD1d-αGalCer complex accumulation with Th2 cell-type cytokine-biasing analogs suggested a reduced requirement for endosomal loading as a general property of such glycolipids. This was confirmed with a functional assay to compare presentation of each analog by APCs expressing either wild-type mCD1d (mCD1d.WT) or a cytoplasmic tail deletion mutant (mCD1d.TD) deficient in endosomal trafficking and localization (Jayawardena-Wolf et al., 2001) (Figure 4B). With A20 B lymphoma cells expressing mCD1d.WT, αGalCer-C26:0 and -C24:0 were significantly more potent at activating iNKT cells compared to all Th2 cell-type cytokine-biasing analogs. In contrast, the opposite was observed with A20 cells expressing mCD1d.TD. This inversion of relative potencies was consistent with a requirement for endosomal loading of mCD1d by αGalCer-C26:0 and -C24:0 and with a general lack of this requirement for analogs that preferentially induced Th2 cell-type cytokine responses.

### Requirement for Lipid Transfer Factors

**Figure 5:**
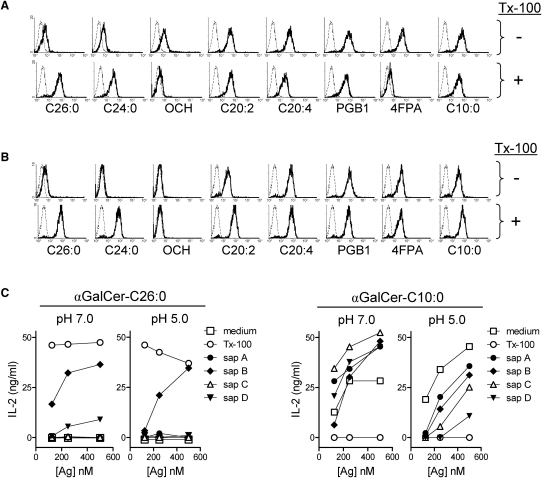
Lack of Detergent-like Cofactor Dependence of Th2 Cell-Type Cytokine-Biasing αGalCer Analogs (A) Glycolipids were incubated for 24 hr at 37°C with murine CD1d proteins in the absence (top row) or presence (bottom row) of 0.05% Triton X-100. The resulting complexes were assembled as tetramers and used for staining mouse iNKT hybridoma DN3A4.1-2. The dashed line histograms show background staining with unloaded tetramers, and the bold histograms show staining with glycolipid loaded tetramers. (B) The same analysis as in (A) except with human CD1d tetramers, which were also used for DN3A4.1-2 staining. (C) Glycolipids at various concentrations were incubated with immobilized mCD1d protein for 2 hr in pH 7.0 or pH 5.0 buffers with no additives (medium) or in the same buffers containing the indicated purified recombinant saposins (10 μg/ml) or Triton X-100 (0.05%). After washing, DN32.D3 iNKT hybridoma cells were added and supernatant concentrations of IL-2 were determined after an additional 14 hr of incubation. All results shown (A, B, and C) were representative of at least two independent experiments.

The efficient loading of CD1 molecules with lipids such as αGalCer-C26:0 is known to involve cofactors that enhance the transfer of lipids from micelles or membranes into the antigen-binding groove of the CD1 protein. The best known of these cofactors are resident intracellular lipid binding and transfer proteins including those belonging to the saposin family, GM2 activator proteins, and Niemann-Pick type C1 and C2 proteins (Zhou et al., 2004; Sagiv et al., 2006; Yuan et al., 2007; Schrantz et al., 2007). Most of these are known to have detergent-like activities that facilitate solubilization of lipids and their exchange between hydrophobic binding sites. In an in vitro model for this activity of endosomal lipid transfer proteins, we investigated the impact of detergent (0.05% Triton X-100) on the loading of αGalCer analogs onto purified mCD1d or hCD1d tetramers (Figures 5A and 5B). With mCD1d, there was a clear enhancing effect of Triton X-100 on the formation of tetramers that bound to iNKT cell TCRs when αGalCer-C26:0 or -C24:0 were used as ligands, whereas loading of all six Th2 cell-type cytokine-biasing analogs was optimal in the absence of detergent (Figure 5A). Largely similar findings were obtained with hCD1d tetramers (OCH failed to form hCD1d tetramers with iNKT cell-binding avidity under any conditions) (Figure 5B). Overall, this consistent lack of detergent dependence for loading of CD1d by all Th2 cell-type cytokine-biasing analogs was a further unifying feature of such compounds and pointed to differences in aqueous solubility of αGalCer analogs as an important determinant of their effects on iNKT cell activation.

To extend these observations to a more physiological model, we tested the effects of purified recombinant saposin A, B, C, and D proteins on loading of αGalCer analogs. In an in vitro loading system with recombinant mCD1d and a functional readout with iNKT cell hybridoma responses, we found that loading of αGalCer-C26:0 was dependent either on the presence of detergent (0.1% Triton X-100) or on the presence of a purified saposin (Figure 5C). Saposin B was strongly active in this regard, with minimal or no activity for saposins A, C, or D. This enhancing effect of saposin B was observed at neutral pH and remained strong and specific under acidic conditions approximating the endosomal environment in which saposins normally function. In marked contrast, all four saposins showed only modest enhancement of loading with αGalCer-C10:0 at neutral pH and actually reduced mCD1d loading at acidic pH.

### Lipid Raft Localization of αGalCer-CD1d Complexes

**Figure 6:**
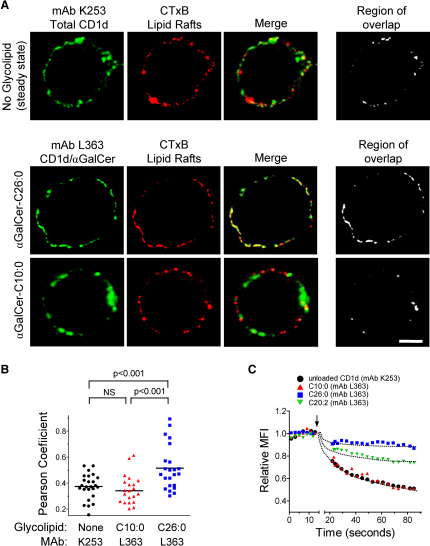
Plasma Membrane Lipid Raft Association of CD1d-αGalCer Complexes (A) Top row shows steady state localization of total cell-surface mCD1d on JAWS II cells (anti-mCD1d mAb K253 (green), and cholera toxin B subunit (CTxB) followed by clustering with anti-CTxB antiserum (red). Areas of overlap in the merged image are shown in the black-and-white image on the right (region of overlap). In the two lower rows, JAWS II cells were incubated for 18 hr with the indicated glycolipids and then stained with CD1d-αGalCer complex-specific mAb L363 (green) and CTxB (red). Merged and region of overlap images show marked colocalization of mCD1d-αGalCer-C26:0 with GM1-enriched lipid rafts, as opposed to minimal overlap for mCD1d-αGalCer-C10:0 complexes. The scale bar represents 10 μm. Results shown for individual cells were typical of >20 cells analyzed for each parameter with or without incubation with the indicated glycolipids. (B) Quantitation of colocalization shown in (A) for multiple randomly selected individual cells. Pearson correlation coefficient for the distribution of pixel densities for K253 or L363 fluorescence compared to cholera toxin B-specific fluorescence are plotted for each individual cell analyzed. Values for p were determined by one-way ANOVA. (C) Flow cytometry assay for αGalCer-mCD1d complex association with detergent-resistant membrane microdomains. JAWS II cells were incubated with or without the indicated glycolipids for 18 hr and stained with mAbs K253 or L363. Baseline fluorescence levels were recorded for 15 s, after which Triton X-100 was added to 0.1% (arrow) and recording was resumed for another ∼60 s. Each symbol indicates mean fluorescence intensity (MFI) averaged over 1 s of recording. MFI values were normalized to the mean baseline levels for each sample during the initial 15 s of recording. Results are representative of three experiments.

Recent studies highlight the localization of a fraction of CD1d proteins to detergent insoluble plasma membrane microdomains (i.e., lipid rafts) and suggest that this may have functional relevance for iNKT cell activation (Lang et al., 2004; Park et al., 2005; Peng et al., 2007). We assessed the potential involvement of lipid rafts in controlling the cytokine bias of iNKT cell activation by comparing the extent of raft colocalization of mCD1d complexes forming in APCs pulsed with αGalCer-C26:0 versus -C10:0 (Figure 6A). Consistent with the findings of Peng et al., a relatively small fraction of total surface mCD1d expressed by JAWS II cells colocalized with lipid rafts, which were identified by the binding of cholera toxin B subunit (CTxB) to the GM1 gangliosides enriched in these microdomains (Kenworthy et al., 2000). In contrast, imaging of mAb L363 binding to cells that had been incubated with αGalCer-C26:0 showed strong colocalization with CTxB staining, indicating localization predominantly in lipid rafts. This was not observed for L363 staining of cells incubated with αGalCer-C10:0; this staining showed extremely limited colocalization with CTxB staining indicating exclusion from GM1-enriched lipid rafts (Figures 6A and 6B).

Another property of lipid rafts is their resistance to solubilization by nonionic detergents due to their enrichment in cholesterol, sphingomyelin, and saturated acyl chains (Pike, 2008). To assess the localization of αGalCer-mCD1d complexes in detergent insoluble microdomains, we used a flow-cytometry-based assay that quantitates the resistance of lipid-raft-associated transmembrane proteins to extraction by low concentrations of Triton X-100 (Gombos et al., 2004a; Peng et al., 2007). Using this approach (Figure 6C), we observed that a substantial fraction (∼70%) of total surface mCD1d (identified with mAb K253) could be extracted from the cell surface within 90 s by 0.1% Triton X-100, consistent with a major nonraft localizing cohort of mCD1d. Similarly, the majority of L363 staining resulting from preincubation of the cells with αGalCer-C10:0 was eliminated by a brief exposure to 0.1% Triton X-100, indicating that mCD1d complexes containing this glycolipid were mainly localized to detergent soluble nonraft domains of the plasma membrane. Complexes formed with αGalCer-C20:2 also showed significant extraction by detergent in these experiments, although less complete than for αGalCer-C10:0. In striking contrast, complexes of mCD1d containing αGalCer-C26:0 were extremely resistant to detergent extraction (∼90% remaining after 90 s).

**Figure 7:**
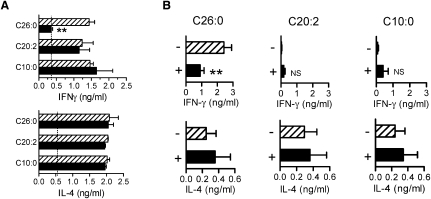
Functional Consequences of Membrane Lipid Raft Association of Glycolipid-Loaded CD1d Molecules (A) C57BL/6 BMDCs were pulsed for 18 hr with the indicated glycolipids, washed, and incubated for 15 min in medium alone (hatched bars) or medium containing 10 mM MβCD (solid bars). Cells were then fixed (1% paraformaldehyde) and used for stimulating autologous splenocytes for 24 hr, after which supernatant concentrations of IFN-γ and IL-4 were measured. Dotted lines indicate the background levels of cytokines in cultures with BMDC pulsed with vehicle only. Means and standard deviations for triplicate cultures are shown, and results are representative of three experiments. ^∗∗^p < 0.01 (ANOVA, Bonferroni post-test). (B) Effect of MβCD on in vivo presentation of αGalCer analogs. Splenic DCs were pulsed ex vivo with the indicated glycolipids for 18 hr and then incubated for 15 min in medium without (−, hatched bars) or with 10 mM MβCD (+, solid bars). After extensive washing, the cells were injected i.p. into naive C57BL/6 mice (10^6^ cells/mouse). Serum was assayed for IL-4 at 2 hr and for IFN-γ at 24 hr postinjection. Data shown are means and standard deviations for pooled data from two experiments that had three mice per group. ^∗∗^p < 0.01 (ANOVA, Bonferroni post-test); NS, not significant (p > 0.05).

These results strongly indicated that αGalCer analogs differ profoundly with respect to presentation by lipid-raft-associated or nonraft-associated mCD1d molecules, and such a difference can be envisioned to have major effects on the strength and quality of iNKT cell activation (Park et al., 2005). To assess functional consequences of raft localization of CD1d-glycolipid complexes, we analyzed the effects of methyl-β-cyclodextrin (MβCD) treatment on the iNKT cell stimulatory capacity of CD1d-glycolipid complexes in vitro and in vivo (Figure 7). MβCD substantially disrupts lipid raft microdomain structures by extracting cholesterol from the outer leaflet of the plasma membrane and reduces the raft association of plasma membrane CD1d molecules (Park et al., 2005). Treatment of DCs pulsed with αGalCer-C26:0 with MβCD markedly reduced their ability to stimulate IFN-γ production by splenocytes and had no effect on stimulation of IL-4. This effect was not observed for in vitro stimulations with αGalCer-C20:2- or αGalCer-C10:0-pulsed DCs, which were unaffected by MβCD (Figure 7A). A similar result was obtained for in vivo iNKT cell stimulation after injection of MβCD-treated DCs (Figure 7B). These results implied that cholesterol-rich plasma membrane lipid rafts were highly relevant for the mixed cytokine response to αGalCer-C26:0 but were dispensable for iNKT cell responses to Th2 cell-type cytokine-biasing agonists, consistent with their lack of presentation by lipid-raft-associated CD1d molecules.

## Discussion

Structurally modified forms of αGalCer have been explored in recent years as a strategy for more selectively activating specific functions of iNKT cells to exploit their therapeutic potential (Miyamoto et al., 2001; Schmieg et al., 2003; Miyake and Yamamura, 2005; Yu et al., 2005; Forestier et al., 2007). The current study addresses the fundamental mechanisms that cause certain structural analogs of αGalCer to activate the strong, preferential production of Th2 cell-type cytokines by iNKT cells that is associated with anti-inflammatory or tolerizing effects. Our studies extend other work centered mainly on the truncated sphingosine analog known as OCH, which emphasized the extremely low affinity or avidity of this ligand for iNKT cell TCRs (Oki et al., 2004). The weak binding of OCH-loaded CD1d molecules to the iNKT cell TCR, confirmed in the current study, suggested that an affinity threshold model could account for the Th2 cell-type cytokine bias and overall anti-inflammatory effects that are observed after activation of iNKT cells with this αGalCer variant (McCarthy et al., 2007).

However, our findings argue against an affinity threshold model as the principle mechanism accounting for the properties of many or most Th2 cell-type cytokine-biasing iNKT cell agonists. As demonstrated in an earlier study (Forestier et al., 2007) and extended here, these analogs showed a remarkable range of avidities for mouse or human iNKT cell TCRs. Structural studies of CD1d-glycolipid complexes also suggest that altered functional outcomes of iNKT cells to N-acyl variants are unlikely to be due purely to direct effects on TCR affinity (Zajonc et al., 2005; Koch et al., 2005; Borg et al., 2007). These studies reinforce the view that alterations in the sphingoid base or acyl chain of αGalCer are mostly hidden from the TCR contact surface by their positioning within the A' and F' pockets of the CD1d lipid-binding groove. Although modeling suggests that a structural rearrangement of the TCR contact surface may be a result of incomplete filling of the A' pocket by the truncated sphingoid base of OCH (McCarthy et al., 2007), the high avidity of at least some Th2 cell-type biasing N-acyl variants of αGalCer for iNKT cell TCRs argues against such effects as a universal explanation for the altered cytokine responses. Thus, mechanisms other than or in addition to variations in signaling based simply on TCR affinity must underlie the altered cytokine responses stimulated by certain αGalCer analogs.

An alternate mechanism supported by our data relates to how these compounds become associated with CD1d in APCs. Our findings did not strongly support the previously proposed hypothesis that presentation by different APC types accounts for qualitative differences in iNKT cell responses (Bezbradica et al., 2005; Im et al., 2006). Instead, what emerged strongly from our studies was a consistent tendency for Th2 cell-type-biasing glycolipids to be presented with rapid kinetics and without a requirement for intracellular loading of CD1d, apparently as a result of their ability to rapidly associate with CD1d molecules directly at the cell surface. This was in marked contrast to αGalCer-C26:0, which underwent intracellular loading and was presented more slowly. It is noteworthy that a similar finding has been reported recently for αGalCer-C20:2 in studies that used novel phage display-derived antibodies with TCR-like specificity against the hCD1d/αGalCer complexes (Denkberg et al., 2008), suggesting the potential relevance of our observations to human iNKT cell responses.

The lack of a requirement for detergent or for a lipid transfer protein (e.g., saposin B) as an accessory factor for glycolipid loading was also consistently observed for Th2 cell-type-biasing glycolipids, and we speculate that this was related directly to their ability to rapidly load surface-expressed CD1d molecules. The chemical structures of αGalCer analogs with pronounced Th2 cell-type cytokine-biasing effects are variable, but all of these are characterized by substantial shortening of their aliphatic chains or the inclusion of polar substitutions such as double bonds or oxygen atoms. These modifications increase the overall polarity and reduce the hydrophobicity of these glycolipids relative to compounds giving mixed cytokine responses like αGalCer-C26:0 and -C24:0, or the even more hydrophobic Th1 cell-type cytokine-biasing C-glycoside variant of αGalCer-C26:0 (Schmieg et al., 2003; Fujii et al., 2006).

Our demonstration that αGalCer analogs can differ markedly with regard to the extent to which they are presented by lipid-raft-associated CD1d molecules provides an attractive mechanism to explain the stimulation of biased cytokine responses by particular glycolipids. It is likely that the forced intracellular loading of a compound such as αGalCer-C26:0 leads to the organized transport of glycolipid-CD1d complexes into cholesterol-rich lipid rafts, which also are known to facilitate recruitment into immunological synapses along with a variety of other molecules involved in T cell activation (Gombos et al., 2004b). Conversely, direct loading of CD1d molecules on the surface of the cell results in exclusion from such lipid rafts and therefore promotes a very different type of iNKT cell activation. Such lipid-raft-dependent alternative activation, although novel for iNKT cells and CD1d-dependent antigen recognition, has previously been described in studies of MHC class II presentation that have remarkable parallels with our observations on CD1d. For example, it has been shown that a substantial fraction of plasma membrane MHC class II molecules are concentrated in cholesterol-rich lipid rafts and tetraspannin microdomains (Poloso and Roche, 2004). Analogous to our findings with CD1d-presented glycolipids, peptide antigens loaded onto MHC class II molecules in intracellular compartments are presented preferentially in rafts and favor the stimulation of Th1 cell-type responses by conventional CD4^+^ T cells. In contrast, peptides loaded exogenously at the cell surface are presented mainly by molecules outside of rafts and show a tendency to stimulate Th2 cell-type responses (Buatois et al., 2003).

Although our experiments used synthetic lipid agonists to show how ligand structure may regulate the quality of iNKT cell responses, the results may provide insight into how natural endogenous and foreign lipid ligands are presented and sensed by the CD1d-iNKT cell axis. Thus, the preferential loading of glycolipid antigens with relatively short or polyunsaturated alkyl tails onto cell-surface CD1d proteins may represent a constitutive process of normal self -recognition that biases the immune system toward tolerance or Th2 cell-type cytokine responses in the absence of an infectious agent. Interestingly, other recent data indicate that glycolipids with this type of structure are not only more efficient at loading directly onto surface CD1d but are also actively excluded from forming stable complexes with endosomal CD1d at low pH (A. Bendelac, personal communication). Such findings are mirrored by our observation that recombinant saposins have the ability to unload αGalCer-C10:0 from CD1d at pH 5.0, but not at neutral pH. Taken together, these studies are evidence for a finely tuned antigen-presenting system in which the sorting of structurally divergent glycolipids onto different cohorts of CD1d molecules is a key mechanism for regulating the balance between tolerogenic and proinflammatory functions of iNKT cells.

## Experimental Procedures

### Mice, Cell Lines, and Reagents

Female C57BL/6 and BALB/c mice were purchased from the Jackson Laboratory and housed under specific pathogen-free conditions. All procedures were approved by the Institutional Animal Use Committee. Murine iNKT hybridomas DN3A4-1.2 (Vα14Vβ8.2) and DN3A4-1.4 (Vα14Vβ10) and A20 B lymphoma cells transfected with wild-type or tail-deleted mouse CD1d proteins were provided by M. Kronenberg (La Jolla Institute for Allergy and Immunology, San Diego, CA, USA). Murine iNKT hybridomas N38.2H4 (Vα14Vβ7) and DN32.D3 (Vα14Vβ8.2) were provided by K. Hayakawa (Fox Chase Cancer Center, Philadelphia, PA, USA) and A. Bendelac (University of Chicago), respectively. The C57BL/6.*Trp53*^−/−^-derived dendritic cell line JAWS II was from the American Type Culture Collection. Murine cells were cultured in RPMI-1640 with 10% fetal calf serum, 100 mM HEPES, and 50 μM 2-mercaptoethanol (complete medium). Human iNKT cell clones and monocyte-derived DCs were produced and cultivated as described (Exley et al., 1997). Monoclonal antibodies specific for mouse CD3ɛ (clone 145-2C11, APC-conjugated), NK1.1 (PK136, APC), CD11c (HL3, APC), and F4/80 (BM8, FITC) were purchased from BD Biosciences. Anti-mouse CD19 (clone 6D5, SPRD) was purchased from Southern Biotech. Monoclonal antibodies to human CD3 (OKT3, FITC) and to human iNKT invariant TCRα chain CDR3 region (6B11, PE) were from BD Biosciences. Anti-LAMP-1 monoclonal antibody (clone 1D4B, Alexa Fluor 488) was from Santa Cruz Biotechnology. The mCD1d-αGalCer complex-specific mAb L363 and mCD1d-specific mAb K253 were produced in our laboratory (Yu et al., 2007). Monoclonal antibody pairs specific for human and mouse cytokines were used in capture ELISAs as previously described (Im et al., 2006; Forestier et al., 2007). Glycolipids used in this study were synthesized and solubilized for in vivo injection or addition to cell culture media as described (Ndonye et al., 2005; Yu et al., 2005; Forestier et al., 2007).

### In Vitro and In Vivo Activation of iNKT Cells

For in vitro stimulation, murine iNKT hybridomas at 5 × 10^4^ cells/well in 96-well plates were stimulated with an equal number of either JAWS II cells or transfected A20 cells in complete medium with glycolipids for 12 hr at 37°C, and levels of murine IL-2 secretion were determined. For some experiments, APCs were preincubated with glycolipids for 12 hr or fixed with paraformaldehyde (1% for 2 min at RT) prior to the addition of iNKT cell hybridomas. For in vitro stimulation of murine splenic iNKT cells, splenocytes from BALB/c or C57BL/6 mice were plated at 5 × 10^5^ cells per well in complete medium in 96-well plates and stimulated with glycolipids for 48 hr at 37°C. Subsequently the levels of IL-4, IL-13, and IFN-γ in culture supernatants were quantified by capture ELISA. For assays with methyl-β-cyclodextrin (MβCD) treatment of APCs, we prepared splenic DCs with a CD11c^+^ cell isolation kit from Miltenyi Biotech from mice that had been injected s.c. 14 days previously with 10^5^ Flt3-ligand-secreting B16 melanoma cells to increase the yield (Mach et al., 2000). Purified CD11c^+^ cells were cultured in complete medium for 18 hr with glycolipids (100 nM) as indicated, washed, and incubated at 37°C for 15 min in medium with or without 10 mM MβCD (Sigma-Aldrich) and subsequently fixed (1% paraformaldehyde) and cultured in 96-well plates at 2 × 10^5^ cells/well with autologous splenocytes (4 × 10^5^ cells per well). Supernatants were harvested 24 hr later for measurement of cytokines. In vivo activation of iNKT cells by i.p. glycolipid injection (4 nmoles) of female C57BL/6 mice and measurement of serum cytokines was done as previously described (Forestier et al., 2007). For determination of MβCD effects on in vivo presentation, CD11c^+^ purified splenic DCs from C57BL/6 mice injected s.c. 14 days previously with 10^5^ Flt3-ligand-secreting B16 melanoma cells were cultured in complete medium with 100 nM of glycolipids for 18 hr, washed, and incubated for 15 min in RPMI-1640 with or without 10 mM MβCD. After extensive washing, the cells were injected i.p. into naive C57BL/6 mice (10^6^ cells/mouse), and blood was collected at 2 hr and 24 hr for analysis of serum cytokine levels.

### CD1d Tetramer Assembly and Flow Cytometry

Recombinant CD1d proteins were produced and assembled into tetramers for staining as previously described (Forestier et al., 2007). To obtain optimally loaded tetramers for iNKT cell staining, we loaded mCD1d with αGalCer-C26:0 and -C24:0 in the presence of 0.05% Triton X-100 (Sigma-Aldrich) and loaded mCD1d with other glycolipids without Triton X-100. For loading of hCD1d, 0.05% Triton X-100 was included with αGalCer-C26:0, -C24:0, and -C20:4, whereas other glycolipids were loaded without Triton X-100. For surface staining of αGalCer/mCD1d complexes, murine splenocytes or JAWS II cells were incubated with 250 nM glycolipids for various times and then 0.2 μg/ml mAb L363 (Alexa Fluor 647 or biotin conjugated) was added directly to the culture for an additional 30 min at RT. In some experiments, additional fluorochrome-conjugated antibodies specific for leukocyte subpopulations were added for the last 15 min of incubation. All samples were analyzed with the FACSCalibur flow cytometer (BD Biosciences).

### In Vitro CD1d Loading Assay

Recombinant murine CD1d proteins at 4 μg/ml in 50 μl PBS were coated on the surfaces of microtiter plate wells for 2 hr at 37°C and then washed with PBS for removal of unbound proteins. Subsequently, various concentrations of glycolipids in 0.1 M sodium phosphate buffer (pH 7.0) or 0.1 M sodium citrate buffer (pH 5.0) containing 150 mM NaCl were incubated with or without 0.05% Triton X-100 or recombinant purified human saposins A, B, C, or D at 10 μg/ml for another 2 hr at 37°C. Saposins were produced and purified as described (Yuan et al., 2007). After extensive washing with PBS and media, 5 × 10^4^ murine iNKT hybridoma DN32.D3 cells were added in 200 μl of complete medium and incubated at 37°C for 14 hr. Supernatants were analyzed for murine IL-2 by capture ELISA.

### Confocal Microscopy and Lipid Raft Analysis

JAWS II cells were incubated at 37°C with 100 nM glycolipids for various times as indicated. For surface staining, live cells were harvested and washed extensively with PBS, resuspended in PBS + 1% BSA + 0.1% NaN_3_, stained on ice with 10 μg/ml Alexa Fluor 647-conjugated mAb L363 for 30 min, and lastly washed extensively and fixed with 4% paraformaldehyde. Cells were washed with PBS and settled onto poly-L-lysine-coated coverslips for 30 min and mounted with ProLong Gold with DAPI mounting medium (Invitrogen). For intracellular staining, JAWS II cells grown on glass coverslips were fixed in 4% paraformaldehyde, permeabilized with 0.05% Triton X-100 for 5 min, and stained with 10 μg/ml Alexa Fluor 647-conjugated L363 and FITC-conjugated anti-LAMP-1. Coverslips were washed and mounted. Confocal images were obtained with a Leica SP2 AOBS confocal microscope (Leica Microsystems). For colocalization of αGalCer/mCD1d complexes with ganglioside GM1-enriched plasma membrane lipid rafts, JAWS II cells were incubated for 18 hr at 37°C in complete medium without or with 100 nM of glycolipids. Cells were then washed with PBS, resuspended in PBS + 1% BSA + 0.1% NaN_3_, and stained on ice with the 10 μg/ml of Alexa Fluor 647-conjugated mAb L363 or mAb K253 plus cholera toxin B subunit (Invitrogen) for 30 min. After washing once with PBS, cells were incubated with Texas Red-conjugated rabbit anti-cholera toxin B, fixed, and mounted for confocal microscopy. We performed quantitative analysis of colocalization by selecting 20–25 random cells in each group and determining a Pearson correlation coefficient for the distribution of pixel densities for K253 or L363 fluorescence compared to cholera toxin B-specific fluorescence. Assessment of mCD1d or αGalCer/mCD1d complex association with detergent resistant membrane microdomains by flow cytometry was carried out with accordance to a published protocol (Gombos et al., 2004a). JAWS II cells were incubated in complete medium with or without indicated glycolipids for 18 hr, harvested, washed, and resuspended in PBS with 1% FCS. After staining with Alexa Flour 647 conjugates of mAbs K253 or L363, cells were resuspended at 10^6^/ml in PBS with 1% FCS and analyzed for fluorescence with the kinetic mode for acquisition with time as a variable with Cell Quest software and the FACSCalibur (BD Biosciences). After recording baseline fluorescence for 15 s, Triton X-100 was added to a final concentration of 0.1% with brief (∼1 s) vortexing to mix, and acquisition of fluorescence level in kinetic mode was continued for another 60 s.

## Supplemental Data

Document S1. Five Figures
