# Supplementary material for: Kinetics and Cellular Site of Glycolipid Loading Control the Outcome of Natural Killer T Cell Activation
Source: Immunity. 2009 Jun 19;30(6):888–98. doi: 10.1016/j.immuni.2009.03.022 (PMC2719696; doi:10.1016/j.immuni.2009.03.022)
Supplement: Document S1. Five Figures [file mmc1.pdf]

Supplemental Data

Kinetics and Cellular Site of Glycolipid Loading Control the Outcome of Natural Killer T Cell Activation

Jin S. Im, Pooja Arora, Gabriel Bricard, Alberto Molano, Manjunatha M. Venkataswamy, Ian Baine, Elliot S. Jerud, Michael F. Goldberg, Andres Baena, Karl O. A. Yu, Rachel M. Ndonye, Amy R. Howell, Weiming Yuan, Peter Cresswell, Young-tae Chang, Petr A. Illarionov, Gurdyal S. Besra, and Steven A. Porcelli

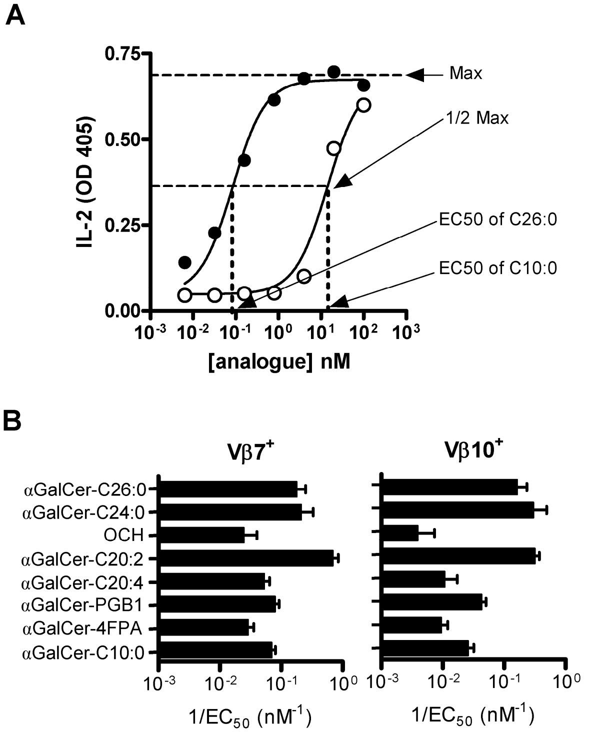

Figure S1. Determination of glycolipid potency using iNKT cell hybridomas

(A) In the example shown, Vβ10$^+$ mouse iNKT cell hybridoma DN3A4-1.4 was cultured with RMA-S.CD1d antigen presenting cells and varying concentrations of αGalCer-C26:0 (filled symbols) or αGalCer-C10:0 (open symbols) as described in Experimental Procedures. IL-2 release (expressed here as OD_{405}) was measured by ELISA of supernatants harvested 18 hours later. The EC_{50} (effective concentration 50%) values

for each glycolipid were determined as the concentration of glycolipid giving 50% of the maximal OD_{405} value based on extrapolation to the X-axis.

(B) Mouse iNKT cell hybridomas, N38.2H4 (Vβ7^{+}) and DN3A4.1-4 (Vβ10^{+}) were stimulated with each of the αGalCer analogues at a range of concentrations using JAWS II cells as APCs, and the levels of IL-2 production were analyzed. The potencies of glycolipids are shown as $1/EC_{50}$. Values plotted are means ± standard deviations for triplicate cultures. The data are representative of three experiments.

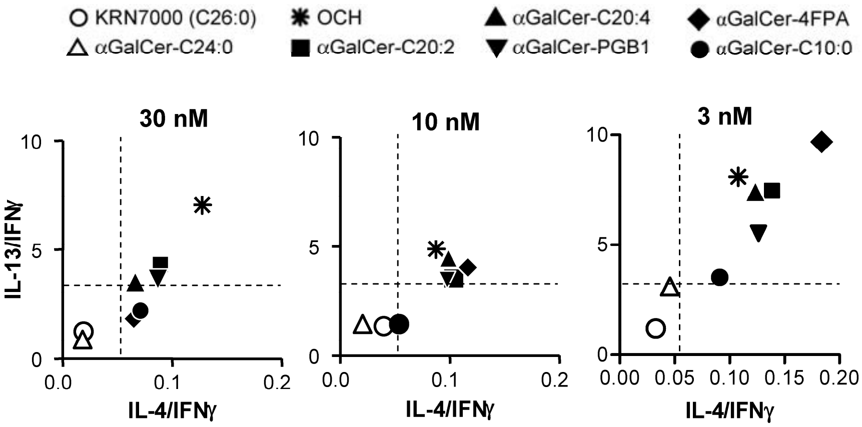

Figure S2. (A) Splenocytes from BALB/c mice were stimulated with glycolipids at 30, 10 or 3 nM for 48 hours, and supernatants were analyzed for selected Th1 cell- (IFNγ) or Th2 cell (IL-4 and IL-13) type cytokines. The ratios of IL-4 to IFNγ (x-axis) were plotted against the ratios of IL-13 to IFNγ (y-axis). The data are representative of three experiments.

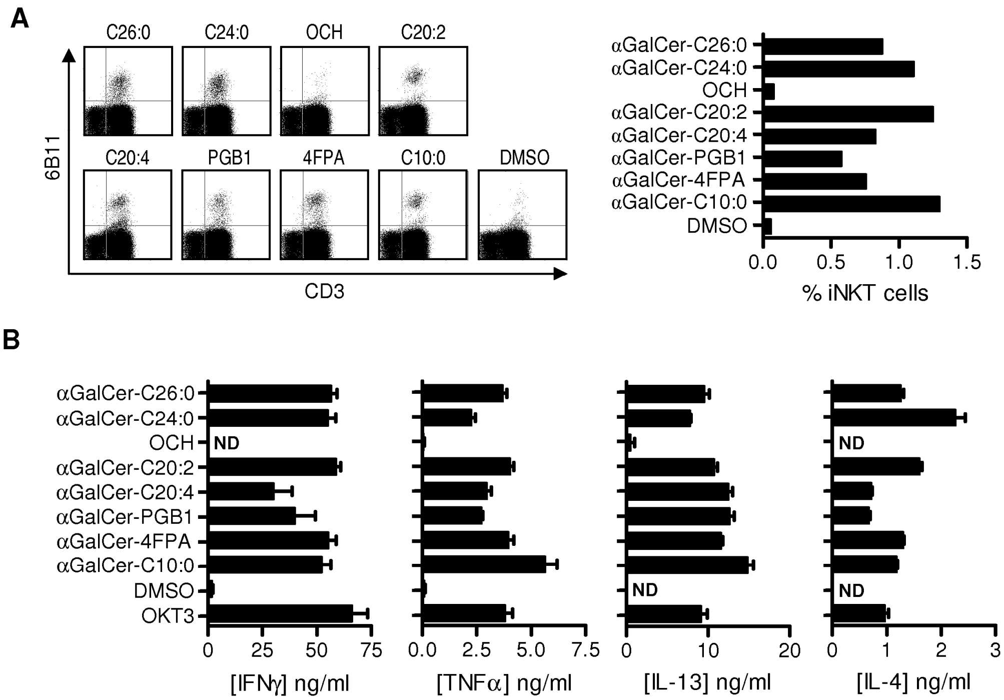

Figure S3. Responses of human iNKT cells to Th2 cell-type cytokine biasing αGalCer analogues

(A) For *in vitro* expansion of iNKT cells, 2 million human PBMC per well in 96 well plates were stimulated with 100 nM of glycolipids in the presence of IL-2 (60 IU/ml) and IL-7 (5 ng/ml). At day 7, cultures were harvested and analyzed by FACS using 6B11 (specific for the TCRα chain of iNKT cells) and anti-CD3 mAbs. Significant expansion of human iNKT cells (expressed as a percentage of total viable lymphocytes) was observed with all glycolipids with the exception of OCH. Similar results were obtained in three independent experiments from 6 different donors.

(B) Cytokine production by a $CD4^+$ human iNKT clone (HDD11) stimulated with human monocyte-derived dendritic cells pulsed with αGalCer analogues. For *in vitro* activation of human iNKT cells, 5×10^4 cloned iNKT cells per well were stimulated with 2.5×10^4

human monocyte-derived DCs in the presence of glycolipids for 48 hours. Supernatant levels of cytokines were measured by capture ELISA. Values plotted are means ± standard deviations for triplicate cultures. ND, not detected. Similar results were obtained using two other human iNKT cell clones, which included clones with $CD4^-CD8^-$ (double negative) and $CD8\alpha^+$ phenotypes. Interestingly, while all glycolipid analogues except for OCH were strongly stimulatory, a clear Th2 cell-type polarization of cytokine production was not evident for any of these analogues in assays using human iNKT cell clones. Thus, the overall pattern of cytokine production in these experiments was consistent with the previously proposed view that Th2 cell-type cytokine bias following iNKT cell activation with certain glycolipid ligands results mainly from reduced transactivation of IFNγ production by NK cells or other bystander cells, rather than from flexibility in the cytokine profiles of the iNKT cells themselves.

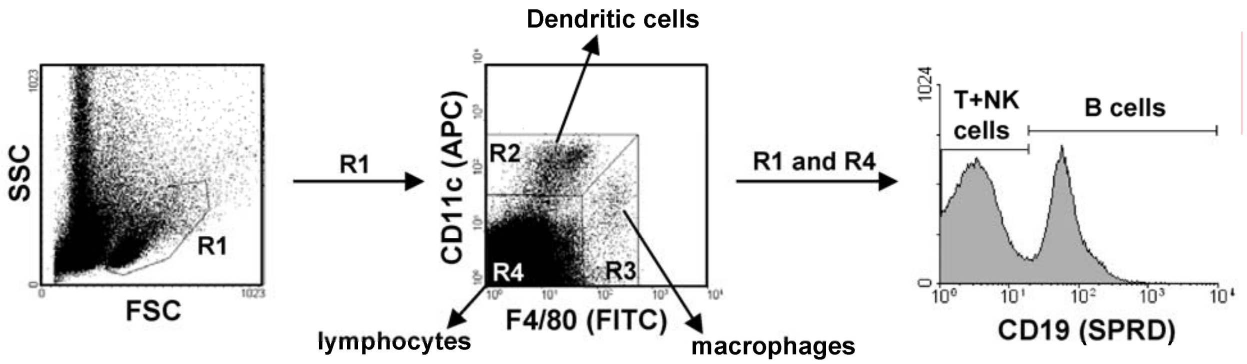

Figure S4. Mouse splenocytes were cultured with either vehicle only (0.02% DMSO), or with each of the αGalCer analogues at a concentration of 100 nM. Cells were harvested after 1 hour or 19 hours of incubation, and stained with biotinylated mAb L363 or biotinylated anti-CD1d mAb 1B1 plus streptavidin-PE, and with mAbs specific for CD11c, CD19 and F4/80. Shown is a representative example of the gating strategy used to assess staining with mAb L363 (specific for murine CD1d/αGalCer complexes) on DCs ($CD11c^{+}$, $F4/80^{-}$, $CD19^{-}$), B cells ($CD11c^{-}$, $F4/80^{-}$, $CD19^{+}$) and the predominately T cell plus NK cell fraction ($CD11c^{-}$, $F4/80^{-}$, $CD19^{-}$).

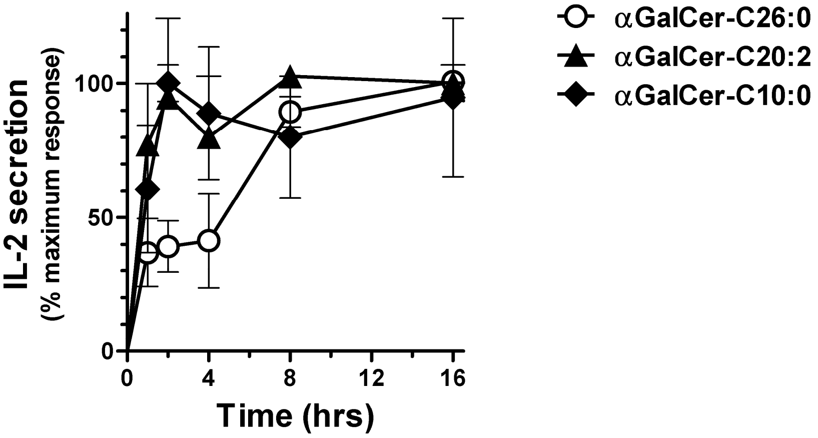

Figure S5. 2.5 X 10^4 BMDCs were pulsed with αGalCer analogues at 100nM concentration. The cells were incubated with glycolipids for different periods of time as shown on the X-axis, and then fixed with 1% PFA for 1min at RT. After extensive washing with PBS, 5 X 10^5 DN32.D3 iNKT hybridoma cells were added. The supernatant was analyzed for IL-2 after 12 hours of incubation. Mean and standard deviations for triplicate values are shown, and results were representative of three similar experiments.
